# Anticipated regret to increase uptake of colorectal cancer screening in Scotland (ARTICS): study protocol for a randomised controlled trial

**DOI:** 10.1186/1471-2458-13-849

**Published:** 2013-09-16

**Authors:** Ronan E O’Carroll, Robert JC Steele, Gillian Libby, Linda Brownlee, Julie A Chambers

**Affiliations:** 1 Psychology, School of Natural Sciences, Stirling University, Stirling FK9 4LA, UK; 2 Surgery and Molecular Oncology, Ninewells Hospital, University of Dundee, Dundee DD1 9SY, UK; 3 Scottish Bowel Screening Centre, Kings Cross Hospital, Clepington Rd, Dundee DD3 8EA, UK

**Keywords:** Colorectal cancer, Screening, Anticipated regret, Health locus of control, ‘Ick’ factor

## Abstract

**Background:**

Colorectal cancer is the second leading cause of cancer deaths in the UK. Screening is key to early detection. The Scottish programme of colorectal cancer screening is running successfully, and involves all adults aged between 50 and 74 years being invited to post back a faecal sample for testing every 2 years. However, screening uptake is sub-optimal: for example rates for the period November 2009 to October 2011 ranged from just 39% for males living in the most deprived areas to 67% for least deprived females. Recent research has shown that asking people to consider the emotional consequences of not participating in screening (anticipated regret) can lead to a significant increase in screening uptake.

**Methods/Design:**

We will test a simple anticipated regret manipulation, in a large randomised controlled trial with 60,000 members of the general public. They will be randomly allocated to one of 3 arms, no questionnaire, control questionnaire or anticipated regret questionnaire. The primary outcome will be screening test kit return. Results will also be examined by demographic variables (age, gender, deprivation) as these are currently related to screening kit return.

**Discussion:**

If this anticipated regret intervention leads to a significant increase in colorectal cancer screening kit returns, this would represent a rare example of a theoretically-driven, simple intervention that could result in earlier detection of colorectal cancer and many more lives saved.

**Trial registration:**

Current Controlled trials: ISRCTN74986452

## Background

### The problem

Scotland has one of the highest incidences of colorectal cancer (CRC) in the world (43.6 per 100,000 in men, 28.4 per 100,000 in women) and, as in many Western countries, the disease represents the second most common cause of cancer death [1]. Screening is the key to early detection. However, colorectal screening participation varies considerably across countries, and is rarely above 60% [2]. There is also a marked social gradient in participation, with higher social classes often showing double the participation of lower social classes. The importance of this social gradient is further highlighted by the fact that poorer survival following the diagnosis of colorectal cancer is associated with lower socioeconomic status (SES) [2].

A national programme of CRC screening using a guaiac faecal occult blood test (FOBT) is now underway across Scotland [3]. This programme is running successfully and involves all adults aged 50–74 being invited to take their own faecal sample and return it by post for testing every 2 years. The clear social gradient is also strikingly apparent in Scotland, with response rates from low SES groups (particularly males) currently well below 50% [3]. A meta-analysis of screening using FOBT showed a 23% reduction in CRC mortality in participants [4]. Similarly, a recent matched cohort study in Scotland found that screening led to a reduction in overall colorectal cancer mortality by 10%, and in the population that participated, this increased to 27% [5]. Thus the potential benefit of the FOBT screening test clearly depends on the level of uptake, and increasing this is a priority.

### Theoretical background

Although there is recognition that screening uptake rates are low, little work has attempted to identify the key factors which could maximise uptake. This research addresses this gap by drawing on contemporary psychological theories which help explain human behaviour. While many people may approve of FOBT in principle, they may not return their own test kit. The gap between *intention* (e.g. wanting or expecting to use FOBT in the future) and *behaviour* (i.e. actually returning the FOBT test pack) can best be understood by employing a dual-process model in which human behaviour is shaped by two systems. The first is a reflective, rational, goal-oriented system driven by our values and intentions. It requires cognitive capacity, and most traditional approaches to health promotion depend on engaging this system [6]. Often based on providing information, these approaches are designed to educate and alter beliefs and attitudes and motivate people via the prospect of future benefits. At best, these approaches have a modest effect on changing behaviour. The second is an automatic, affective system that requires little or no cognitive engagement, and is more driven by immediate feelings and emotions [6]. Recent work has suggested that health self-care decisions are often not primarily a consequence of rational evaluation of evidence, but may be more directly influenced by emotional/visceral affective beliefs and attitudes (“gut feelings”). In this project we will test whether activating the emotion of anticipated regret leads to a significant increase in FOBT screening uptake.

#### ***Anticipated regret (AR)***

Regret is a negative cognitive-based emotion that is experienced when we imagine that the present situation could have been better had we acted differently. It is also possible to *anticipate* regret and thus act to, or prepare to, avoid actually experiencing this unpleasant emotion. It has been shown that over and above the traditional attitudinal components of influential social cognitive theories such as the Theory of Planned Behaviour (TPB), anticipated regret (AR) adds significantly to the prediction of intentions and prospective health behaviours [7]. To take an illustrative example from the screening literature, Sandberg and Conner [8] invited three groups of women for cervical screening: a control group, a group sent a TPB questionnaire and a group who were asked to complete a TPB questionnaire, and also answer two AR questions on a Likert-style 7 point scale; “If I did not attend for a cervical smear in the next few weeks I would feel regret”, and “If I did not attend for a cervical smear in the next few weeks, I would later wish I had”. In the intention to treat (ITT) analysis (which included those who did not return the questionnaire), screening attendance was 21%, 26% and 26% respectively (i.e. simply sending out a questionnaire increased attendance by 5%). For those who completed and returned the questionnaire (i.e. were definitely exposed to the intervention) attendance rates were 21%, 44% and 65% respectively. This is a quite remarkable effect, given the simplicity, low cost and low intensity of the intervention.

The research to date indicates that subtle AR interventions can significantly increase the likelihood of intention being translated into behaviour, e.g. increasing screening uptake, condom use, exercise, and weight loss [9]. Subtly increasing the prominence of AR in the decision-making process emphasises the aversive emotional consequences of not taking action, and the desire to avoid the aversive feeling of regret then motivates people to translate their positive intentions into action. Essentially, AR strengthens behavioural intentions and binds the person to action, because failing to act is associated with aversive emotions. The present study aims to determine whether a simple AR intervention significantly increases FOBT screening uptake.

#### ***Mere measurement effect***

There is also growing evidence for behavioural change induced by simply completing a relevant questionnaire – the “mere measurement effect”. Put simply, this proposes that asking someone to complete any questionnaire about a behaviour increases the likelihood that they will subsequently engage in that particular behaviour [10-12]. We will therefore attempt to control for this possible mere measurement effect by running an RCT with 3 arms, (1) no questionnaire, (2) general health belief questionnaire and (3) general health belief questionnaire with two additional AR questions. This will allow us to test whether AR leads to a significant increase in FOBT screening uptake, over and above the mere measurement effect. In RCT arm 2, participants will be asked to complete a questionnaire assessing general beliefs about health (but critically not AR). The health belief scale we will employ is the Multidimensional Health Locus of Control Scale [13]. This scale measures the individual’s belief that health (and health outcomes) are determined primarily by: (a) themselves (internal) (b) chance or fate and (c) powerful others (e.g. doctors). Importantly, individuals who believe their health outcomes are largely predetermined by fate (e.g. high chance locus of control) are significantly less likely to engage in health protective strategies, such as FOBT screening [14].

#### ***Emotional barriers to FOBT***

Finally, a key potential psychological barrier to collecting and returning one’s own stool sample is the emotion of disgust. Jones at al. [15] recently assessed patient-reported barriers to CRC screening in over 6,000 participants. In relation to FOBT screening, they found that two major perceived barriers were commonly reported: “I do not want to handle my stool” and “I do not want to keep my stools on a card in my house”. Furthermore, Chapple et al. [16] conducted a qualitative study on barriers to FOBT, and found that many participants reported disgust at the idea of handling stools, together with concern about posting their samples in the mail. Accordingly, we will also test whether those individuals who report higher levels of disgust (the so-called “ick factor”) [17], are significantly less likely to return their FOBT sample.

### Relevant evidence from the CRC screening literature

Recently, Gregory et al. [18] concluded that: “Beliefs about the benefits of screening and the barriers to completing CRC screening are key predictors of participation, and provide a focus for intervention programs”. It is important to note, however, that much of the relevant CRC screening literature is focused on the much more invasive flexible sigmoidoscopy (FS), rather than our specific focus, FOBT.

Wardle et al. [19] conducted an intervention trial comparing a psycho-educational booklet versus control in a trial aimed at increasing participation in FS screening in the UK. Importantly, the development of their intervention booklet was influenced by regret theories. Resultant FS screening attendance was significantly higher in the intervention group (53.5%) versus the control (49.9%). Power at al. [20] subsequently found that AR was better at predicting intention to attend, rather than actual attendance for FS. This finding is at variance with the large body of evidence which has shown that when people anticipate regret if they contemplate failing to carry out a behaviour, they are more likely to act on their intentions [9]. However, attending for FS is also clearly a bigger resource requirement than returning a FOBT screening test by post. Power et al. [20] concluded that despite AR predicting intention, social factors such as low SES and difficult lives may play an important role in acting as barriers to implementing intentions.

#### ***Social Inequalities in FOBT uptake***

Whitaker et al. [21] extended this work by studying the relationship between low SES, social cognitive variables and FS screening attendance. They focused on the construct of “consideration of future consequences” (CFC), a construct that has clear similarities to AR. An example of a questionnaire item tapping CFC is “I’m prepared to make sacrifices now to benefit in the long run”. Both CFC and AR are examples of temporal self-regulation models [22]; both include consideration of the future (anticipation) and a strong affective component (benefits versus regrets). Whitaker et al. [21] also included a perceived benefits scale which included items that clearly tap AR, e.g. “If I don't go, I might later wish I’d been tested”. Whitaker et al. [21] found a clear SES gradient in FS attendance, and that SES differences in CFC contributed to SES differences in the perceived barriers and benefits of screening, which, in turn, contributed to differences in FS screening attendance. They proposed that interventions that take consideration of future consequences into account could promote greater socio-economic equality in screening participation. It is conceivable that high SES individuals are more trained to evaluate facts or evidence, whereas low SES participants may use emotion more to guide decision-making. Thus, our planned emotion-based AR intervention may plausibly help act to reduce SES differences in FOBT uptake.

While it may seem that our simple AR intervention (inviting participants to rate the degree of regret they may feel if they do not return their FOBT test kit) is a minor one, in a recent systematic review, Baron et al. [23] concluded that minimal cue interventions can be effective at increasing cancer screening behaviours, including FOBT. Furthermore, more intensive, tailored interventions have not proven more effective. For example, Smith et al. [24] tested a comprehensive decision aid (including booklet and DVD), which resulted in a 16% *lower rate* of FOBT completion! Thus the available evidence from the CRC screening literature to date would seem to support further rigorous evaluation of theory-based, minimal intensity interventions.

### Pilot studies

We have recently conducted a large, randomised controlled trial of the efficacy of a simple pre-notification letter embedded within the Scottish national colorectal cancer-screening programme [25]. In this study, 60,000 adults aged 50–74 years were randomly allocated to one of 3 conditions: standard invitation (no pre-notification), simple pre-notification letter posted 2 weeks before the test pack, and pre-notification letter plus a more detailed information booklet. Uptake was significantly higher with both the pre-notification letter (59.0%) and the letter plus booklet (58.5%), compared with the usual no-prior notification method of invitation (53.9%) [25]. Importantly, the increase was seen for males and females of *all* age groups and *all* deprivation categories, including the groups with the highest and lowest levels of uptake, i.e. least deprived females (letter 69.9%, usual invitation 66.6%) and most deprived males (42.6% and 36.1%). These results demonstrate that simple interventions can increase uptake in members of *all* deprivation categories [25]. However, despite this clear evidence that pre-notification significantly improved uptake, 30-64% of people sent FOBT kits plus the pre-notification letter still did not return them [25]. As the Scottish Intercollegiate Guidance Network (SIGN) have just recommended that “Population screening for colorectal cancer using the guaiac FOBT should continue in the Scottish population until further evidence on other modalities is available” [1], any increase in return rates of screening kits would help maximise the benefits of this programme.

In a different context, we have also shown that the simple AR manipulation that we propose using here led to a significant increase in intention to become an organ donor [17] and self-reported organ donor registration [26], and we are currently conducting an evaluation of the efficacy of an AR intervention on UK National Health Service Blood and Transplant verified organ donor registration [27]. The current AR intervention study in colorectal cancer screening will test whether a similar intervention significantly increases screening uptake in people from all social deprivation categories.

### Aims

We aim to test whether a simple AR manipulation, sent along with the standard FOBT pre-notification letter, will lead to a significant increase in the uptake of FOBT colorectal cancer screening in Scotland.

### Research questions

1. Does a brief, theory-based AR intervention lead to a significant increase in the uptake of FOBT colorectal cancer screening in Scotland?

2. Is the effect observed equally across genders and social deprivation levels?

3. Is the effect a general consequence of the “mere measurement effect” or is it a specific consequence of AR?

4. Is uptake influenced by participants’ health beliefs, in particular anticipated regret, disgust, intention, perceived benefit and health locus of control?

## Methods/Design

**Figure 1:**
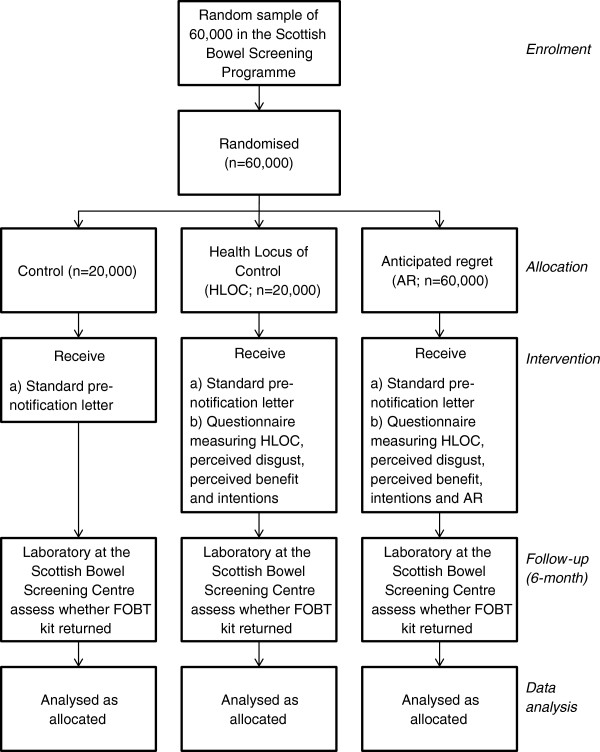
CONSORT diagram of study design.

In this study we aim to manipulate AR, by asking participants to reflect on the emotional consequences of not returning their FOBT screening kit, in an attempt to increase FOBT screening uptake. We propose a simple 3-arm randomised controlled trial (RCT), (1) no questionnaire control (2) HLOC questionnaire control and (3) AR questionnaire. The CONSORT diagram is shown in Figure 1.

### Setting

A single-centre trial based at the Scottish Bowel Screening Centre in Dundee.

### Ethical approval

Ethical approval has been granted by Tayside NHS Board, East of Scotland Research Ethics Committee (REC ref. no. 12/ES/0092).

### Recruitment

Following the method of Libby et al. [25], participants will be a randomly recruited representative sample of all participants in the national colorectal screening programme in Scotland. Participants will be sampled from all residents aged 50–74 years who have a Community Health Index Number (a unique patient identifier) that is associated with an NHS Board. As a result of the findings of Libby et al. [25], the programme now sends all eligible people a standard pre-notification letter 2 weeks before being sent a FOBT by post, which they are required to complete at home and then return to the laboratory at the Scottish Bowel Screening Centre in Dundee for analysis. Our questionnaires will be included along with this pre-notification letter.

#### ***Inclusion criteria***

All patients currently included in the Scottish Bowel Screening Programme are eligible for this study.

#### ***Exclusion criteria***

There are no exclusion criteria.

### Informed consent

Written informed consent is not being sought from participants in the current study. Participants who are allocated to the questionnaire arms will be sent an information sheet saying that we are studying the effects of attitudes towards health and bowel screening, and how they influence FOBT returns. It will be stressed that completion of the questionnaire is totally independent of the decision to return the FOBT kit, and that all responses will be anonymised and confidential. Participants in the control arm will be sent the standard pre-notification letter, as per current practice, which does not require informed consent. If we asked for informed written consent from the two questionnaire arms, we would be treating them differently from the control arm, which may confound any differences found between groups (as those giving informed consent may be more likely to return their screening kit), therefore invalidating the results of the study. This approach was also used in our previous study [25]. It is justified in the current study for the following reasons: 1) no harm will come to participants; 2) it would be impractical for this research to be carried out if we had to obtain informed consent from all participants, as the control arm are receiving no intervention; 3) the data we are seeking to use (i.e. regarding return of FOBT kits) is already available to screening staff at the Scottish Bowel Screening Centre and will be completely anonymised before merger with the questionnaire data; and 4) the potential benefits to participants outweigh the costs. We obtained full UK NHS IRAS ethical approval for this approach (Tayside NHS Board, East of Scotland Research Ethics Committee; REC ref. no. 12/ES/0092).

### Design

We will adopt a simple, between-groups, three-arm (no questionnaire control, HLOC control questionnaire or AR questionnaire), prospective RCT design, following Libby et al. [25]. A large, nationally representative, random sample of the Scottish general public who are invited to participate in the national screening programme will be sampled via post. Participants who are allocated to the questionnaire arms will be told that we are studying the effects of attitudes towards screening.

#### ***Control***

Participants in the control arm will be sent the standard pre-notification letter, according to current practice.

#### ***HLOC Intervention***

Those randomly allocated to the HLOC group will also be sent the pre-notification letter plus the 18-item HLOC scale [13]. HLOC participants will also be asked to rate their perceived disgust (ick factor) and perceived benefit of returning their FOBT using modified versions of the ick-factor scale and perceived benefit scales from O’Carroll et al. [17], as well as rating their intention of returning the FOBT test, all using simple 1–7 Likert-type scales. Participants will be asked to return this brief questionnaire in a stamped addressed envelope that will be provided. We predict that high scores on chance HLOC will predict lower return rates, as this taps a fatalistic view of health and health outcomes.

#### ***AR Intervention***

Those allocated to the AR group will also be sent the pre-notification letter and will be asked to complete the same HLOC/ick/perceived benefit questionnaire as the HLOC group with 2 additional AR questions. Following Sandberg and Conner [8], the first of these additional questions will be placed as the very first question of the survey (“If I did not complete and return my test kit I would later feel regret”) and the second will be placed immediately preceding the final question measuring intention to return the kit (“If I did not complete and return my test kit, I would later wish I had”). In order to make the two questionnaires identical in length, the HLOC questionnaire will have 2 filler questions added in the same location as the AR questions.

### Sampling and randomisation

Sampling will be computer-generated within the Scottish Bowel Screening Centre IT system, which governs the National FOBT screening programme, and which identifies when individuals are to be invited to participate. The IT system electronically generates the appropriate mailing packages to be sent out to each individual. For this study, simple random sampling will be used to allocate individuals to control, HLOC or AR in a 1:1:1 ratio. Following Libby et al. [25], the randomization and allocation sequence will be computer generated by the IT staff, completely independently of the researchers. The process of generating and mailing the FOBT pre-notification letters is fully automated, and is currently handled by a large, not-for-profit, mail-handling company. A data file containing contact details for the pre-notification letters is sent to the mail-handling company on a daily basis. The questionnaires will be added to the pre-notification letters by this company at the time of mailing, therefore blinding the researchers to the allocation of the intervention to individuals. Two additional variables will be added to the usual FOBT coding system: a) a field representing the relevant arm of the study (no questionnaire control = 1, HLOC = 2, AR = 3), and b) a unique identifier to be printed on each pre-notification letter/questionnaire which will then be used to record-link the data with each individual’s subsequent FOBT return.

The questionnaires will be returned to the Division of Psychology at the University of Stirling (in the stamped addressed envelopes that will be provided), where the data will be subsequently scored and entered into the study database by the Research Fellow (JC). The questionnaires do not contain any personally identifying information. Questionnaires will be stored in a locked filing cabinet, and all electronic information will be stored on secure network computers for a period of 10 years, in line with University Policy. Data integrity of questionnaire data will be enforced by valid value and range checks at the time of data entry.

Social deprivation will be categorized using the Scottish Index of Multiple Deprivation (SIMD) which identifies small area concentrations of multiple deprivation across Scotland based on income level, employment, health, education, skills and training, housing, geographic access and crime. SIMD deprivation, age, and information on current and previous FOBT returns will be provided for all individuals sent the pre-notification letter, via the unique identifier, by the Information Services Division (ISD) within the National Health Service, Scotland. Hence, no personally identifiable information will be required for the purposes of this research.

### Strategies to maximise questionnaire return rate

In an attempt to ensure the highest response rate possible, we will implement, where feasible, design recommendations from recent empirical investigations on enhancing response rates in this type of field-based study. These strategies have recently been helpfully summarised in a Cochrane review [28] and include, for example: user-friendly questionnaire layout, short questionnaire, personalising the cover letter to each individual, use of coloured ink, emphasis on confidentiality, stamped (not franked) return envelopes and University Sponsorship.

### Primary outcome

Our primary outcome variable is return of the completed FOBT test kit to the central laboratory at the Scottish Bowel Screening Centre, within 6 months of the kit being sent out. Secondary outcomes will include the HLOC, AR, ‘Ick’ factor (perceived disgust), perceived benefit of completing the test kit and intention to complete the test kit. The HLOC is a well-used and validated measure which has shown good reliability.

### Analysis

Our primary analysis will be chi-square tests to explore the proportion of respondents who return the test kits within 6 months as a function of the 3 arms (control, HLOC or AR). Logistic regression will be used to examine screening uptake by treatment arm, controlling for potential between-arm differences in age, gender and social deprivation, whether a reminder letter was sent, number of previous FOBT returns, and previous failures to return. Our primary analysis will be on an ‘Analysed as Allocated’ basis, i.e. including all participants randomised to treatment arms, whether they return a questionnaire or not. Secondary analysis will be conducted on the questionnaire respondents (HLOC and AR groups only), to test the mediating role of intention, AR, health locus of control, disgust and perceived benefit in influencing FOBT returns.

### Power calculations

Following Libby et al. [25], a sample of 60,000 subjects (20,000 randomized to the usual pre-notification letter group, 20,000 randomized to the HLOC Group and 20,000 to AR group) would enable an increase in FOBT uptake of between 3% and 5% to be detected in all social deprivation groups with 80% power at the 5% level.

### Evaluation

The effects of the intervention will be evaluated in all participants via measurement of the primary and secondary outcome variables listed above.

#### ***Process evaluation***

As this study is based on a one-off questionnaire no process evaluation is required.

### Timetable

This is a 20-month project. Months 1–6 will be spent designing, piloting and finalising the exact layout of the questionnaires and updating the IT system with the variables required for the current research. During months 5–6 we will also finalise the mailing procedures and obtain PAC (Privacy Advisory Committee) approval for the data linkage. The questionnaire packs will be posted out to participants commencing in month 7. The Scottish Bowel Screening Centre estimate that approximately 2,500 pre-notification letters are sent out each day, thus it will take approximately 5–6 weeks to send out 40,000 questionnaires (treatment arms 2–3), plus 20,000 pre-notification letters (treatment arm 1).

Participants can return their FOBT test kit at any point over the following 6 months; therefore we will have a 6-month follow-up period. During months 8–14 the questionnaire returns will be scored as they are returned, and entered into the SPSS study spreadsheet, and the analytic strategy will be finalised. The data extract will be carried out at the end of month 14. Months 15–18 will be spent collating the FOBT returns with the questionnaire scores, and performing the logistic regression and mediation analyses. In months 19–20 we will draft scientific papers, conference presentations and the Chief Scientist Office final report.

## Discussion

It is estimated that a 5% increase in FOBT screening uptake could translate into approximately 11 additional cancers diagnosed per 100,000 of the target population. If a simple AR intervention leads to a significant increase in colorectal screening uptake, this would represent a rare example of a theoretically-driven, relatively simple psychological intervention that could result in earlier detection of colorectal cancer and many more lives being saved. This project has the full support of both the Scottish Bowel Screening Programme Board and the Detect Cancer Early Programme. If the current trial proves a clear advantage of the AR condition, it has the potential for implementation in the future National screening programme, e.g. by adding the AR questions to the pre-notification letter. If the results of the trial are positive, they will be communicated immediately to Scottish Bowel Screening Programme Board, who will then advise the Scottish Government Department of Health on implementation.

## Competing interests

The authors declare that they have no competing interests.

## Authors’ contributions

RO’C conceived the study. All the authors were directly involved in the discussions that led to the grant application and/or the writing of this study protocol. All authors are grant holders and all read and approved the final version of the study protocol.

## Pre-publication history

The pre-publication history for this paper can be accessed here:

http://www.biomedcentral.com/1471-2458/13/849/prepub
